# Systematic review of the health and societal effects of medication organisation devices

**DOI:** 10.1186/s12913-016-1446-y

**Published:** 2016-07-06

**Authors:** Steven James Watson, Clare Frances Aldus, Christine Bond, Debi Bhattacharya

**Affiliations:** Department of Psychology, Fylde College, Lancaster University, Bailrigg, Lancaster LA1 4YF UK; School of Health Sciences, Edith Cavell Building, University of East Anglia, Norwich Research Park, Norfolk, NR4 7TJ UK; Centre of Academic Primary Care, University of Aberdeen, Foresterhill, Aberdeen, UK; School of Pharmacy, University of East Anglia, Norwich Research Park, Norfolk, NR4 7TJ UK

**Keywords:** Compliance aid, Medication organiser, Multi-compartment device, Adherence, Cost, Pill organiser

## Abstract

**Background:**

Suboptimal medication adherence is a significant threat to public health and resources. Devices that organise weekly doses by time and day are commonly used to reduce unintentional non-adherence. However, there is limited evidence to support their use. This systematic review was conducted to evaluate current evidence for their efficacy, safety and costs.

**Methods:**

A pre-defined search of electronic databases from inception to January 2013 augmented with hand-searching was conducted. No limits were placed on publication date. Studies that compared organisation devices used by patients administering their own medication with standard medication packaging regardless of study design were eligible for inclusion. Studies that solely explored dispensing aspects of organisation devices were included whether or not they compared this to standard care. Screening of articles for inclusion and data extraction were completed independently by two reviewers with disagreements resolved by discussion. Outcomes were categorised into impact on health, medication adherence, healthcare utilisation, dispensing errors, supply procedures and costs. Risk of bias was also assessed.

**Results:**

Seventeen studies met the inclusion criteria. Health outcomes were investigated in seven studies of which three reported a positive effect associated with organisation devices. Medication adherence was reported in eight studies of which three reported a positive effect. Three studies reported health care utilisation data but overall results are inconclusive. No optimal dispensing or supply procedures were identified. Economic assessment of the impact of organisation devices is lacking. All studies were subject to a high risk of bias.

**Conclusions:**

Evidence regarding the effects of medication organisation devices was limited, and the available evidence was susceptible to a high risk of bias. Organisation devices may help unintentional medication non-adherence and could improve health outcomes. There is a strong need for more studies that explore the impact of such devices on patients, and an equally pressing need for studies that explore the impacts on healthcare services.

**Trial registration:**

This systematic review is registered with PROSPERO (Registration number CRD42011001718).

## Background

An estimated 25 % to 50 % of all patients diagnosed with a chronic disease do not take their medication as prescribed [1, 2]. Some of this non-adherence to medication is intentional, with patients actively choosing to deviate from their prescribed regimen because of their beliefs about their illness and medicines, and their experiences of treatment [3]. However patients frequently cite factors such as forgetting, being too busy, or experiencing a change in their daily routines as the main reasons for their non-adherence [4]. Thus, a significant amount of non-adherence is likely to be unintentional.

Non-adherence to medication poses a significant risk to public health, and is an important issue for policy makers. Non-adherence has been demonstrated to result in poorer health outcomes [5] and is associated with an increase in hospital admissions estimated to cost the UK National Health Service up to £196 million per year [6] and the US $100 billion [7]. Thus simple cost-effective interventions to reduce non-adherence are sought to improve public health and reduce avoidable expenditure in healthcare systems.

One such intervention is a Medication Organisation Device (MOD). There are a wide variety of MODs available but all are based on the same principle; at their simplest they comprise a plastic tray formed of a series of wells often in 7 x 4 format providing 28 consecutive combined doses for a one week period. They are designed to help patients prescribed multiple medicines to remember which of these need to be taken at the different times of day, and facilitate identification of any missed doses [8]. The underlying assumption is that MODs are an *aide memoire* and reduce the burden of complex medication regimens. MODs can also be associated with additional features such as electronic reminder systems which are usually light or sound. Whilst these features may be beneficial, the aim of this review was to report the effects of MODs, not reminder systems.

Despite the wide use of MODs [9] there is limited evidence for their use or guidance on how, when, or by whom they should be used [10]. Previous reviews of the evidence have focussed on the impact of MODs on adherence and health outcomes [10–13], and whilst this is the principal purpose of MODs, there are other potential benefits and disadvantages that are overlooked by focussing on these two outcomes alone.

If MODs successfully increase adherence and improve health outcomes, then it should be expected that they could also be associated with a reduction in the need for more expensive healthcare interventions such as visits to the family doctor or a reduction in hospital and nursing home admissions [5, 14]. Thus the potential economic benefits and wider healthcare implications of MODs should be explored.

As well as the potential benefits, MODs may also introduce risks. Foremost they introduce an additional step in the dispensing process when the pharmacist, patient or carer transfers prescribed medications into the MOD. Thus it is important to review evidence of the effect of MODs on dispensing errors. There are also financial and opportunity costs to healthcare systems incurred by dispensing MODs, such as the acquisition cost of the MOD and the time to fill the MOD. Thus, when considering the utility of MODs compared to usual care, it is important to consider cost effectiveness as well as clinical effectiveness.

The aim of this systematic review was to identify current evidence for simple MODs in any population and for any study design with respect to the following outcomes;

1. Patient health outcomes and quality of life.
2. Patient adherence to medication.
3. Patient healthcare and social services utilisation.
4. Pharmacy dispensing errors.
5. Pharmacy supply procedures and associated costs.

## Methods

This systematic review was conducted in accordance with PRISMA and Cochrane guidelines [15, 16] and the study protocol registered with PROSPERO (Registration number CRD42011001718).

Empirical studies of any design, on the use of MODs were included if they reported any of the following:

- Adherence to medicines (using an objective measure (e.g. pill count or electronic monitoring);
- Health outcomes;
- Health related quality of life;
- Health or social care utilisation;
- Dispensing or administration errors;
- Costs associated with prescribing or medicine supply.

We focus on pill counts and electronic monitoring because these are the gold standard measures of medication adherence, avoid self-reporting bias, and unlike other methods of adherence measurement provide a quantifiable estimate of the magnitude of non-adherence [17].

Studies were excluded if: the intervention incorporated additional reminder systems such as alarms, telephone/SMS messaging, daily medication reminder ‘tick’ charts; there was direct observation of medicine administration by a health care professional; medication was not self-administered; the MOD was used as part of a complex intervention where the independent effect of the MOD on outcomes could not be isolated; the study was not reported in English. There were no limits placed on date of publication.

### Search strategies and information sources

**Fig. 1:**
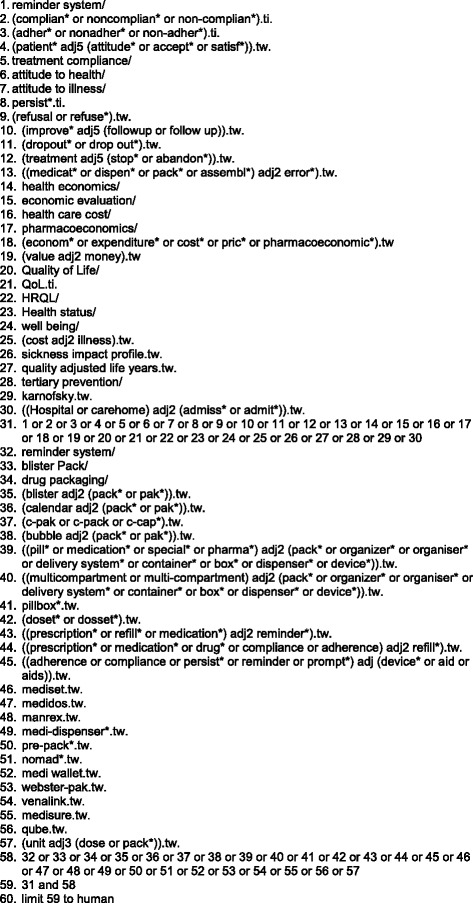
Search terms and syntax for Ovid EmBase (adjusted for other databases)

The following electronic databases were searched from inception to January 2013: The library of the Cochrane collaboration (http://www.cochranelibrary.com/cochrane-database-of-systematic-reviews/), MEDLINE, PsycINFO, AMED, CINAHL, trials listed as complete in Current Controlled Trials (http://controlled-trials.com/) and York Centre for Review and Dissemination databases (http://www.crd.york.ac.uk/crdweb/SearchPage.asp). Thirty different terms referring to adherence, health outcomes, quality of life, health and social care utilisation, errors and costs were combined with 27 referring to drug packaging and drug delivery systems including MOD-specific trade names. Searches were limited to ‘human’ (Fig. 1). The same terms were utilised for each database but with appropriate translation of database specific syntax, for example the symbols and wording required to account for truncation, multiple accepted spellings, adjacency and Boolean operators. The following additional searches were undertaken: hand searching of the reference lists of all identified review articles (including the reference lists of foreign language review articles), of articles whether or not the paper was ultimately included; personal communication with packaging companies and keyword searches in the Google Scholar search engine (http://scholar.google.com).

### Study selection

The titles and abstracts of all articles were reviewed independently against inclusion criteria by two of four reviewers (CA, LC, EP, TB). Disagreement was resolved by discussion. Full texts of potentially relevant papers (*n* = 272) were independently screened by two of three reviewers (CA, EP, TB) using a data extraction tool (see below). When agreement could not be reached, a third reviewer moderated the final decision [16].

### Data extraction

An electronic data extraction form was developed in Excel and piloted to ensure consistency of data extraction across researchers. The form included fields for information about the paper (e.g. authors, year and place of publication); participants and setting of the study (e.g. country, sample size, age range, ethnicity); key study details (e.g. description of methods, study design, setting of research, delivery methods and sampling techniques; intervention and comparator details; outcomes and how they were measured, information to assess risk of study bias, and a summary of results, including summary statistics for meta-analyses. Where possible MODs were described according to seal type (unsealed (reusable)) or sealed (heat- or cold-sealed single-use) and configuration of wells.

Data for all included studies were independently extracted by two reviewers (EP and SW) and any differences were resolved by discussion. For studies with missing data or ambiguities, the corresponding author was contacted for clarification.

### Assessing risk of bias in individual studies

Two reviewers (SW, EP) independently assessed included studies using the Cochrane risk of bias tool [16]. Consensus on the final risk classifications was reached through discussion. Where the study design did not permit randomisation or blinding of assessors this was noted as such designs are at greater risk of bias.

### Data analysis

A priori, a meta-analysis was planned, but due to heterogeneity in the identified studies was not undertaken. Results were tabulated and reported according to the pre-defined outcomes.

## Results

### Study selection

**Fig. 2:**
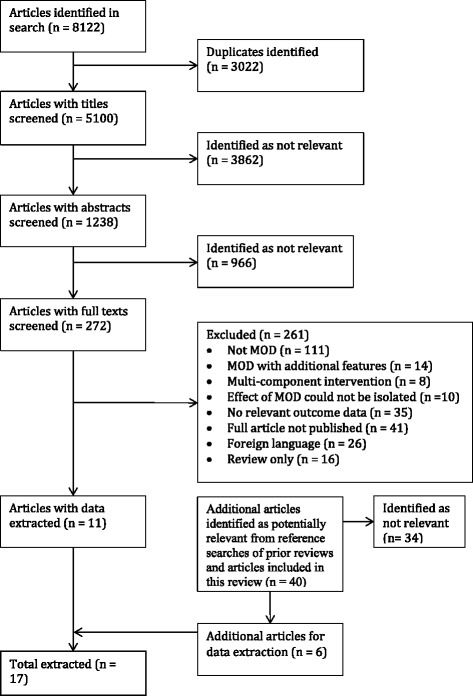
Flow diagram of articles included in the review

The searches identified 8122 potential studies. After removal of duplicates and screening of titles, abstracts and full papers against the inclusion criteria, 17 studies were included. Figure 2 summarises the details of the selection process and reasons for exclusion.

### Overview of studies

A summary of the 17 included studies is shown in Table 1. They were mostly undertaken in either the USA or UK. Ten (59 %) were Randomised Controlled Trials (RCTs), two were prospective cohort studies, two were cross sectional, and one used multiple methods. This final study utilised questionnaires (to collect health outcome and healthcare utilisation) and, a combination of direct observation and audit of pharmacy log books (to collect data on dispensing errors, supply procedures and costs) [18]. Two studies were audits. Nine of the 17 studies were published between 1980 and 1999, and a further eight between 2000 and 2009. No studies measured the impact of MODs on quality of life or social service utilization. No studies declared any conflict of interest.

**Table 1:** Summary of included studies

First Author	Year	Design	Location	Condition	Setting	Age (mean, (range))	Type of MOD (format)	Comparator	MOD filled by	Total n
Becker [20]	1986	RCT	USA	Hypertension	Hospital Outpatient	nr	Sealed. 28 consecutive-dose foil backed blisters (7x4). Packs were perforated so that blisters could be detached.	Standard packaging (bottles)	Automatic device	165
Carruthers [32]	2008	Audit	Australia	Mixed	Care Home	nr	Sealed. 28 consecutive-dose foil backed blisters (7x4). Websterpaks	n/a	Pharmacy	nr *
Crome [27]	1982	RCT	UK	Mixed	Hospital Inpatient	80.29 (68-98)	Unsealed, 28 consecutive-dose compartments (7x4). Dosett®	Standard packaging (bottles)	Unclear	78
Feetam [21]	1982	Prospective	UK	Mental Illness	Community	42.40 (18-68)	Unsealed, 28 consecutive-dose compartments (7x4). Can be divided into single days for convenience. Medidos®	Standard packaging (bottles)	Unclear	10
Huang [23]	2000	RCT	USA	Vitamin supplements in healthy volunteers	Community	58.00 (nr)	Unsealed. Seven consecutive-dose compartments (7x1).	Standard packaging (bottles)	Patient	183
Huang [23]	2000	RCT	USA	Vitamin supplements in healthy volunteers	Community	65.00 (nr)	Unsealed. Seven consecutive-dose compartments (7x1).	Standard packaging (bottles)	Patient	291
Levings [33]	1999	Audit	Australia	Mixed	Community	78.00 (nr)	nr	nr	Various	nr Ɨ
MacIntosh [28]	2007	RCT	Canada	Cancer	Hospital Outpatient	64.00 (42-81)	Unsealed. 14 consecutive-dose compartments (7x2)	Standard packaging (nr)	Researcher	21
McElnay [19]	1992	Cross-section	UK	None	Community	nr	Unsealed, 28 consecutive-dose compartments, various format: Medisystem® (7 of 4x1); Medidos® (7x4), Supercel (7x4, pouches); Dosett®(7x4); Pill Mill® (1x28, wheel-type); Medi-wheel® (4 of 7x1, stacked wheel-type).	n/a	Pharmacist/ technician	6
Petersen [24]	2007	Prospective	USA	HIV	Community	44.00 (38-49)	nr	Standard packaging (bottles)	n/s	269
Rehder [25]	1980	RCT	USA	Hypertension	Hospital Outpatient	51.35 (31-69)	Unsealed, 28 consecutive-dose compartments Mediset®(7x4)	Standard packaging (bottles)	Pharmacy	50
Roberts [18]	2004	Multiple	Australia	Mixed	Community	76.80	nr	n/a	Pharmacy	353
Ryan-Woolley [29]	2005	RCT	UK	Mixed	Care Home	78.80 (67-92)	unclear	Standard packaging (nr)	Pharmacy	62
Simmons [26]	2000	RCT	New Zealand	Diabetes	Community	54.06 (nr)	Sealed. 28 consecutive-dose foil backed blisters (7x4).	Standard packaging (nr)	Pharmacy	68
Skaer [31]	1993	RCT	USA	Diabetes	Pharmacy	51.74	Sealed	Standard packaging (nr)	Pharmacy or researcher	131
Skaer [30]	1993	RCT	USA	Hypertension	Pharmacy	56.49 (nr)	Sealed	Standard packaging (nr)	Pharmacy or researcher	163
Stewart [34]	2001	Cross-section	UK	Mixed	Community	nr	nr	n/a	Nurses	96
Wong [22]	1987	RCT	USA	Mixed	Community	79.00 (66-90)	Sealed.^ 31 non-consecutive-dose foil-backed compartments. Each card serves one daily time point for one month e.g. breakfast.	Standard packaging (bottles)	Pharmacy or researcher	17

Unsealed=resealable/reuseable rigid plastic with sliding or flip-top lids usually filled by patient; Sealed=heat or cold sealed with foil or paper, usually filled in pharmacy; n/a = not applicable; nr = not reported or insufficient detail; * Audit of 2480 patients’ Webster-paks™ at aged care facilities in New South Wales( 6972 packs were audited); Ɨ Study explored errors reported in the Australian Incident Monitoring Study, with analysis based on the 52 incidents involving MODs. ^ 31 compartments per card marked with days of the month. Only one compartment per day. Card marked with e.g. breakfast or dinner. A number of cards needed per patient on any one day

For the studies reporting age, all participants were adults and the median (interquartile) age across these studies was 58.0 (51.5, 78.4) years. Eight studies included participants prescribed medication for the treatment of a chronic condition, and three studies included patients prescribed an antihypertensive. The majority of the studies were in domiciliary settings (*n* = 13). One study [19] examined the effect of MODs on dispensing procedures and no patient data were reported.

Five of the seventeen studies provided little or no information on the type of MOD assessed. Where the type of MOD was specified similar proportions used sealed and unsealed devices. Similar wide variation was demonstrated in terms of comparator (where applicable) and method of fill.

### Risk of bias

Table 2 summarises the risk of bias in included studies. Only three of the nine RCTs reported randomisation details, and none reported blinding of researchers or treatment providers. Other risks of bias identified but not specified in the tool included baseline differences between groups not being accounted for in analyses [18, 20], and minimal information on participants [21, 22] or analytical procedures [21].

**Table 2:** Risk of bias for included studies

		Items from Cochrane risk of bias tool						
Study	Study design	Randomisation procedure	Concealment of allocation	Blinding of assessors	Blinding of treatment providers	Attrition addressed	All outcomes reported	Other risks of bias
Becker [20]	RCT	*?*	*?*	*?*	*x*	*?*	*?*	*x*
Carruthers [32]	Audit	*NA*	*NA*	*NA*	*NA*	*NA*	*?*	✓
Crome [27]	RCT	*?*	*?*	*?*	*?*	✓	*?*	✓
Feetam [21]	Prospective	*NA*	*NA*	*NA*	*NA*	*NA*	*?*	✓
Huang [23]	RCT	✓	✓	*?*	*?*	*?*	*?*	✓
Levings [33]	Audit	*NA*	*NA*	*NA*	*NA*	*NA*	*?*	*x*
MacIntosh [28]	RCT	✓	*?*	*?*	*?*	✓	*?*	✓
McElnay [19]	Cross-section	*NA*	*NA*	*NA*	*NA*	*NA*	*x*	*x*
Petersen [24]	Prospective	*?*	*?*	*?*	*?*	✓	*?*	✓
Rehder [25]	RCT	*?*	*?*	*?*	*?*	*?*	*?*	✓
Roberts [18]	Cross-section, audit	*NA*	*NA*	*NA*	*NA*	*NA*	*?*	*x*
Ryan-Woolley [29]	RCT	*?*	*?*	*?*	*?*	✓	*?*	✓
Simmons [26]	RCT	✓	*?*	*?*	*x*	✓	*?*	✓
Skaer [31]	RCT	*?*	*?*	*?*	*?*	*?*	*?*	✓
Skaer [30]	RCT	*?*	*?*	*?*	*?*	*?*	*?*	✓
Stewart [34]	Cross-section	*NA*	*NA*	*NA*	*NA*	*NA*	*?*	✓
Wong [22]	RCT	*?*	*?*	*?*	*?*	*x*	*?*	*x*

✓ reported/low risk of bias, *x* not reported/high risk of bias, *NA* not applicable*, ?* unclear

### Effect of MODs

The following sections report the clinical and cost effects of MODs; none of the included studies reported cost-effectiveness.

### Health outcomes

Table 3 summarises the impact of MODs on health outcomes for seven studies from six papers (including five RCTs) [18, 20, 23–26]. Three RCTs measured changes in blood pressure [20, 25, 26]. The study of patients with diabetes [26] reported a significant reduction in diastolic blood pressure and HbA1c for the MOD group compared to standard care. In contrast Becker et al. reported no change in diastolic blood pressure, after controlling for baseline/pre-enrolment values and age [20], and Rehder et al. [25] reported no change in systolic blood pressure in the MOD group.

**Table 3:** Impact of MODs on health outcome

Study	Design	Outcome measure	Change in outcome	n MOD	n control	*p*-value
Becker 1986 [20]	RCT	Diastolic blood pressure	β = 1.45	86	85	0.259
Huang 2000a [23]	RCT	Change in Vitamin C serum concentration	-0.9 (mg/dl)	90	94	0.47
		Change in Vitamin E serum concentration	-2.4 (mg/dl)			0.06*
Huang 2000b [23]	RCT	Change in Vitamin E serum concentration	0.9 (mg/dl)	148	149	0.53
Petersen 2007 [24]	Prospective	Viral Load	0.36 mean log_10_ copies/mL. Increased odds of viral load below 400 copies/ml OR 1.91			<0.05*
Rehder 1980 [25]	RCT	Change in diastolic blood pressure	1 mm Hg	25	25	> 0.05
		Change in systolic blood pressure	Not reported			> 0.05
Roberts 2004 [18]	Cross-section	Number of adverse drug reactions (ADRs)	Non-MOD group reported more ADRs (47.79 %) vs non-pharmacist filled MOD group (43.24 %) and pharmacist filled MOD group (32.56 %)			0.022*
		The 14 item Older Americans Resource Scale for Instrumental Activities of Daily Living (OARS-IADL)	Pharmacist filled MOD group had lower ability scores (10.25) vs non-pharmacist filled MOD group (12.70) and non-MOD group (12.34)			0.001*
Simmons 2000 [26]	RCT	Change in diastolic blood pressure	-5.9 mm Hg	36	32	0.0041*
		Change in systolic blood pressure	-1.0 mm Hg			0.89
		Change in HbA1c	-0.8 %			0.026*

*indicates a significance level of *p* < 0.05

n Mod indicates the number of participants in the group utilizing a MOD

n control indicates the number of participants in the group not utilizing a MOD

A prospective longitudinal study of patients prescribed medication for HIV reported a significant improvement in health outcome (reduced viral load) [24] A cross-sectional study of older people prescribed a range of medication in a MOD [18] reported fewer adverse drug reactions when pharmacist-filled MODs were used compared with either MODs filled by the patient, carer/family member, or community nurse, or medication supplied in standard packaging. However a limitation of this comparison was that the patient groups differed in functional ability as assessed by the Older Americans Resource Scale for Instrumental Activities of Daily Living (OARS-IADL) (mean scores were lower for the group receiving MODs filled by pharmacists compared with those filled by a non-pharmacist.

The remaining two studies, both RCTs [23], identified no significant improvement in health outcomes for patients using self-filled MODs.

### Medication adherence

Eight studies (seven RCTs and one prospective longitudinal study, reported the effect of MODs on medication adherence as summarised by Table 4. Adherence was estimated by pill count in all studies; the prospective study used additional electronic monitoring for the control group only [24]. Adherence exceeded 80 % for both the intervention and control group in five of the eight studies. Three studies reported a significant improvement in adherence [22, 24, 25] for the MOD group and three studies failed to identify any difference between the two groups [20, 23, 27]. One study [28] had very high rates of adherence to an oral anticancer medication in both the standard packaging and MOD group, with 18 of the 21 patients in the first group and 17 of the 21 in the latter group having 100 % adherence. The non-randomised study [24] used marginal structural models to estimate the mean difference in percentage of doses taken between patients receiving a MOD and those using standard medication packaging and this identified a 4 % increase in adherence in participants using MODs.

**Table 4:** Effect of MODs on medication adherence

Study	Design	Adherence measure	n MOD	n Control	Adherence MOD	Adherence control	*p*-value
Becker 1986 [20]	RCT	% participants taking > 80 % of doses	86	85	84	75.3	> 0.05
Crome 1982 [27]	RCT	% doses missed	40	38	26.1	26.2	> 0.05
Huang 2000a [23]	RCT	% participants > 90 % of doses	90	94	91	94	Not stated
		Median % of doses taken		94	100	99	0.63
Huang 2000b [23]	RCT	% participants taking > 90 % of doses	148	149	87	93	0.005*
		Median % of doses taken			99	99	> 0.05
MacIntosh 2007 [28]	RCT	% participants taking 100 % of doses	21	21	81	86	Not stated
Petersen 2007 [24]	Prospective	Increase in % doses taken			4.1 %	-	< 0.05*
Rehder 1980 [25]	RCT	% participants taking > 95 % of doses	25	25	89	47	< 0.01*
Wong 1987 [22]	RCT	% doses missed	17	17	2.04	9.17	< 0.01*

*indicates a significance level of *p* < 0.05

n Mod indicates the number of participants in the group utilizing a MOD

n control indicates the number of participants in the group not utilizing a MOD

### Healthcare utilisation

Only three studies [18, 29, 30] examined the effects of MODs on healthcare utilisation. In all three studies the MODs were filled at the pharmacy. No consistent findings were identified; a significant reduction in non-emergency access to healthcare was reported by Roberts et al. [18] in a cross-sectional study. However, non-significant reductions in costs of non-emergency access to physicians (-$19.51) and hospital expenditure (-$22.91) were reported in an RCT by Skaer et al. [30] and a small, significant increase in healthcare utilisation was observed in an RCT by Ryan-Woolley et al. [29].

The effects of MODs on healthcare utilisation are provided in Table 5. Estimates of MOD impact on prescribing costs were provided in two RCT studies with conflicting findings [29, 30]. Skaer et al. using medication possession ratios, reported a significant increase in mean prescription expenditure of $74.09 for patients with diabetes [30] and $48.17 for patients with hypertension [31]. In both cases these extra costs negated savings reported in hospital and non-emergency healthcare access to generate a non-significant overall increase in healthcare costs for patients with MODs compared to controls. Ryan-Woolley et al. reported a significant reduction in the number of prescribed medicines.

**Table 5:** Impact of MODs on healthcare utilisation

Study	Design	n MOD	n Control	Healthcare Utilisation Measure	Healthcare Utilisation MOD	Healthcare Utilisation Control	*p*-value
Roberts 2004 [18]	Cross-section	209	144	Mean no. consultations with a different prescriber	2.02 (pharmacist supplied)	2.41	0.012*
					2.91 (non-pharmacist supplied)		
				Mean no. prescriber consultations in previous two months	2.54 (pharmacist supplied)	3.05	0.03*
					2.05 (non-pharmacist supplied)		
				Mean no. hospital admissions in previous 12 months	1.36 (pharmacist supplied)	0.78	0.001*
					0.56 (non-pharmacist supplied)		
				% patients hospitalised in previous three months	59.54 % (pharmacist supplied)	35.14 %	
					35.14 % non-pharmacist supplied)		
Ryan-Woolley 2005 [29]	RCT	31	31	Mean no. prescriber consultations	1.5	1.3	0.07
				Mean no. prescribed medicines	4.2	4.8	0 .024*
Skaer 1993 [31]	RCT	85	78	Mean healthcare spending (Medicaid archive data)	$13.66 per patient increase compared to control group		> 0.05
Skaer 1993 [30]	RCT	53	78	Mean healthcare spending (Medicaid archive data)	$22.94 per patient increase compared to control group		> 0.05

*indicates a significance level of *p* < 0.05

n Mod indicates the number of participants in the group utilizing a MOD

n control indicates the number of participants in the group not utilizing a MOD

### Dispensing errors

Three studies investigated the frequency of dispensing errors [18, 32, 33]; there was little consistency in findings. All studies discussed the error rate with MODs without reference to any comparison. The study by Carruthers et al, [32] described a nurse conducted audit of the accuracy of filling MODs by nurses. The study included 2480 residents in 42 regional aged care facilities. Errors were identified in 4.3 % (297/6972) of MODs involving 12 % of the residents. The different sources of error are displayed in Table 6. In a smaller study, [18] 190 direct observations by researchers of MODs being filled by pharmacist, dispensary assistant, or pre-registration pharmacy student reported an error rate of 44.7 % of MODs. This compared to only 5.7 % when reported by staff. It was suggested that when observed, the persons filling the MODs made more errors and that the researcher had a more stringent definition of an error. The error types are also displayed in Table 6. Higher error rates were associated with larger facilities (*χ2* = 6.374, *p* = 0.042), longer duration of time spent filling (*r* = 0.342, *p* = 0.004), and interruptions (*r* = - 0.337, *p* = 0.003).

**Table 6:** Summary of the types of errors identified

Study	Design	Error Type	Error rate
Carruthers 2008 [32]	RCT	Omission of a medicine	99/297 (33.3 %)
		Supplying a medicine discontinued by the general practitioner	37/297 (12.5 %)
		Wrong strength	32/297 (10.8 %)
		Incorrect instructions	32/297 (10.8 %)
		Failure to deliver medicines	13/297 (4.4 %)
		Wrong medicine	12.297 (4.0 %)
		Wrong label	7/297 (2.4 %)
		Other/Unknown	65/297 (21.9 %)
		General practitioner error	79/297 (26.6 %)
		Pharmacy error	125/297 (42.1 %)
		Not attributable	93/297 (31.3 %)
Roberts 2004 [18]	Cross-section, audit	Missing tablets	75/190 (39.5 %)
		Extra tablets	46/190 (24.2 %)
		Tablet in wrong position	23/190 (12.1 %)
		Medication changes not made	17/190 (8.9 %)
		Labels incorrect	9/190 (4.7 %)
		Wrong tablet	7/190 (3.7 %)
		Pack/sachet/drug damaged	6/190 (3.2 %)
		Special medications not packed correctly/packed when should not be	4/190 (2.1 %)
		Authority script required	2/190 (1.1 %)
		Wrong colour card	1/190 (0.5 %)

Finally an audit by Levings et al*.* [33] (Australian Incident Monitoring Study) examined the first 12,000 generic incident reports. Reports involving MODs accounted for 0.43 % of all errors (54/12,000) and described 54 separate MOD-related incidents. Of these 54 incidents, 26 (48 %) were either actual (16) or potential (10) errors associated with filling MODs, 16 (29.6 %) were patient errors and 12 (eight actual and four potential) were associated with inappropriate concomitant use of drugs.

### Supply procedures and costs

Four studies estimated the time taken to fill MODs [18, 19, 21, 34]. In a simulated study involving five pharmacists and a pharmacy technician using medicines for five fictitious patients, McElnay and Thompson [19] compared the time taken to fill six different MODs and the perceived ease of filling (Table 7). Pharmacist-ranked ‘ease of filling’ largely matched the ranking for fill time. Even for the MOD which was the quickest to fill, it was estimated that it would take on average seven minutes per patient per month longer than for standard packaging. This estimate was for MODs filled from pre-prepared bottles of medication already removed from the manufacturer’s packaging, and based on multiplying the 105 s taken to fill the (Dosset^TM^) MOD by four (weeks). The study was reported in 1992. At that time manufacturers supplied medicines in bottles or tubs whereas current practice is primarily blister packs. In order to dispense into MODs, transfer of medication is generally therefore from blister packs rather than bottles which adds to the fill-times estimated in 1992. Furthermore, there was no consideration of the time resource required for labelling.

**Table 7:** Summary of MOD filling experience [19]

MOD	Time taken	Perceived ease
	(minutes: seconds)	(1 - favourable to10 - unfavourable)
Dosset	1:45	8.3
Pill Mill	2:27	5
Round tray 4 compartments 7 days		
Medsystem Week Pouch	2:48	7.7
Daily boxes with four compartments in a pouch		
Medidos	2:52	7.8
Stacked wheels 4 compartments 7 days		
Medi-Wheel	3:20	7.5
Stacked wheels 4 compartments 7 days		
Supercel	9:59	1.5
Seven by four pouches on a card		

Roberts [18] using direct observation and audit of log books, identified that the time required to fill a MOD ranged from 3.2 to 8.6 min for large packing operations (supplying 351 – 5000 patients per week) and 14 to 18.5 min for small packing operations (supplying less than 90 patients per week). Time spent checking MODs ranged from 1.13 to 2.13 min for large operations to 3.01 to 8.61 min for small operations. Those filled using automated packing systems took less time to pack (0.99 min) while blister packs (3.34 min) and Dosset™ boxes (10 min) took longer.

Feetam and Kelly [21] found that a Medidos™ MOD took an average of three minutes to fill and 24 min to label in a prospective study.

In a survey of 153 Scottish community nurses, 96 (63 %) reported experience of filling MODs [34]. The estimated average time for a nurse to fill one MOD was 34.2 min. The survey also identified concerns regarding the impact of this workload on nurse schedules, lack of any formal training (most had received no training, 25 % had received informal training) and lack of knowledge about which medicines could be placed in MODs. Nearly 60 % of the nurses felt that pharmacists should fill the MODs.

Two studies examined the costs of using MODs [18, 21]. Feetam and Kelly [21] estimated the cost of supplying Medidos^TM^ MODs for six months to be 0.1 GBP per week compared to 0.21 GBP per week for seven disposable bottles. Similarly labelling was less expensive (0.01 GBP versus 0.04 GBP), as was time spent labelling and filling (0.16 GBP versus 0.24 GBP). Overall this produced an estimated cost of 0.27 GBP per week for a Medidos™ containing seven medicines per day versus seven pill bottles costing 0.49 GBP per week. Roberts [32], however, reported the cost of providing medication in standard medication packaging (usual care) relative to a MOD. The cost of original packaging per year ($942.73) was less than using a MOD ($1859.00 per year).

## Discussion

This systematic review identified 17 studies examining the effect(s) of MODs on health outcomes or supply processes. Unlike previous reviews, this study intended to isolate the effects of MODs in the absence of reminder devices, calendars or other memory aids. Overall study quality was poor, both in terms of research design and execution. Heterogeneity in types of patient, type of MOD and reported outcomes precluded a meta-analysis.

Overall evidence for efficacy of MODs was mixed; whilst there was some suggestion of benefit such as improved adherence, or reduced service utilisation this was not reflected in all studies. This uncertainty regarding MOD effects on pill count measured adherence is not reflected in the review conducted by Mahtani et al.; this reported a pooled effect of significantly greater adherence with MODs relative to control [10]. There are two potential explanations for this difference in outcome. The first is that Mahtani et al. use a wider definition of “pill count” than the present review by including studies such as Skaer et al. [31] that measured adherence using prescription refills. This is an imprecise measure of adherence as it is significantly removed from the act of taking medication taking [35]. The second is that their conclusions are primarily based on their meta-analysis which demonstrated a high level of heterogeneity. The results should therefore be interpreted with caution as estimates can be as much the consequence of differences between studies as any effect of the intervention. The present study identified similarly high levels of heterogeneity thus a narrative review was deemed more appropriate. One reason for a lack of efficacy could be that MODs were not always targeted at patients with an identified need, and in those studies in which patients were targeted, greater benefit was observed [18, 24]. Ideally a study seeking to appraise MODs should select patients demonstrating unintentional non-adherence. Even then MODs represent only one of many potential strategies that could help to reduce non-adherence by facilitating habit-forming strategies [36–38]. Previous reviews have drawn similar conclusions such as Mahatani et al. [10] who state that “there is no single intervention strategy which has been shown to be effective across all patients, conditions and settings”. Unlike the present study, they do not offer any indication of the circumstances that are more likely to be associated with MOD benefits.

Pill count was used to estimate adherence in the intervention group for all studies. Pill count is a pragmatic and widely accepted approach to adherence assessment [3, 17] and despite its limitations regarded as the gold standard when electronic monitoring is not possible [17]. Whilst it is objective, it is based on the assumption that if the medication is not in the container it has been taken by the patient. This is a limitation because patients may deliberately remove and discard tablets in order to disguise non-adherence when under observation [7]. Thus the assumption is only valid if patients are predominantly unintentionally non-adherent. However, in the identified studies, participant non-adherence type was not identified thus there is a risk of overestimating adherence [39]. Pill counts also fail to identify patterns of non-adherence; occasional missed doses and longer breaks from taking medication are not distinguished as only the absolute number of medicines taken is estimated [17, 40]. Such limitations of reporting are not considered by previous reviews [10–13, 41].

Evidence for the utility of MODs in reducing the need for healthcare services such as physician visits or hospitalisation was also mixed. One study found that MODs initiated by pharmacists reduced the number of community physician visits but hospitalisations increased [18]. A second study reported increased community physician visits but a reduction in the number of medications prescribed [29]. It is difficult to attribute causality in these cases. In Ryan-Wooley and Rees [29] the difference is small and potentially due to chance, while in the study reported by Roberts [18] patients initiated on a MOD by a pharmacist scored lower than comparison groups in terms of functional ability and so may have been less able to complete day to day activities. However, and more fundamentally, the cross-sectional nature of Roberts [18] makes causal assumptions especially difficult. Whilst the evidence is far from unequivocal, these two studies do suggest the possibility that MODs increase the need for healthcare utilisation, for example by increasing the risk of adverse drug reactions [41]. It is important that further research be conducted to explore the possibility of this potential hazard [42].

A second cause for concern was the potential for MODs to introduce dispensing errors into patients’ medication regimens. Again evidence is scant; two studies found reported error rates of around 4-6 % [18, 32], but independent researcher checking identified a much higher error rate of over 40 % [18]. There is a clear need to identify both the rate and severity of errors associated with the use of MODs. The error rate may also differ depending on who is filling the MOD; it was notable that one study found that community nurses asked to fill MODs considered themselves unqualified for this task which adversely affected their working schedules [34]. Thus there could be benefit in identifying the most appropriate way to fill MODs that minimises potentially harmful errors while optimising the use of healthcare professionals’ time. Similarly, the economic costs and benefits of staff filling MODs are currently unknown. Only two studies have explored the costs of supplying MODs and provide conflicting evidence for the overall cost effectiveness of doing so [18, 21].

The systematic review methodology adopted followed standard best practice. However, as with all searches it is possible that some papers were missed either through the search process, or because only papers written in English were included. Only eight of the papers reported work conducted since 2000, and the relevance of the older papers may therefore also be queried because of changing contexts, increased understanding of adherence and methodological best practice. Boeni et al. reported a similar observation, noting that reporting quality improved significantly in studies published after the CONSORT statements were published [13].

## Conclusions

There is a dearth of evidence for the use of MODs. There is potential for MODs to improve patient health outcomes and adherence to medication, but this benefit may come with an increased risk of additional healthcare intervention from a general practitioner or even hospitalisation. Currently the evidence base is not available to properly appraise these potential risks or benefits, and given the wide usage of MODs the potential for harm should be a significant cause for concern. There is also a wider need for more thorough appraisal of the economic benefits or otherwise of MODs, including comparing them to other interventions which seek to alleviate unintentional non-adherence. These studies are necessary before MODs can be used optimally.

## Abbreviations

MOD, medication organisation device; OARS-IADL; older Americans resource scale for instrumental activities of daily living; RCTs, Randomised Controlled Trials
